# A Placebo-Controlled, Double-Blind Randomized (Phase IIB) Trial of Oral Administration with HPV16 E7-Expressing *Lactobacillus*, GLBL101c, for the Treatment of Cervical Intraepithelial Neoplasia Grade 2 (CIN2)

**DOI:** 10.3390/vaccines9040329

**Published:** 2021-04-01

**Authors:** Yuji Ikeda, Katsuyuki Adachi, Kensuke Tomio, Satoko Eguchi-Kojima, Tetsushi Tsuruga, Mayuyo Uchino-Mori, Ayumi Taguchi, Atsushi Komatsu, Takeshi Nagamatsu, Katsutoshi Oda, Ai Kawana-Tachikawa, Yukari Uemura, Shizunobu Igimi, Yutaka Osuga, Tomoyuki Fujii, Kei Kawana

**Affiliations:** 1 Department of Obstetrics and Gynecology, Nihon University School of Medicine, 30-1 Oyaguchi-kamimachi, Itabashi-ku, Tokyo 173-8610, Japan; Ikeda.yuji@nihon-u.ac.jp (Y.I.); komatsu.atsushi@nihon-u.ac.jp (A.K.); 2 Department of Obstetrics and Gynecology, Faculty of Medicine, The University of Tokyo, 7-3-1 Hongo, Bunkyo-ku, Tokyo 113-8655, Japan; kadachi-gyn@umin.org (K.A.); k-tomio@umin.ac.jp (K.T.); satokojolly@yahoo.co.jp (S.E.-K.); tsurugatetsushi@gmail.com (T.T.); mayuyo1976@gmail.com (M.U.-M.); ayumikidu246@gmail.com (A.T.); tnag-tky@umin.ac.jp (T.N.); katsutoshi-tky@umin.ac.jp (K.O.); yutakaos-tky@umin.ac.jp (Y.O.); fujiit-tky@umin.org (T.F.); 3 AIDS Research Center, National Institute of Infectious Diseases, 1-23-1 Toyama, Shinjuku-ku, Tokyo 162-8640, Japan; aiktachi@niid.go.jp; 4 Biostatistics Section, Clinical Research Center, National Center for Global Health and Medicine, Tokyo 162-8655, Japan; yukariuemura-tky@umin.ac.jp; 5 Department of Applied Biology and Chemistry, Tokyo University of Agriculture, 1-1-1 Sakuragaoka, Setagaya-ku, Tokyo 156-8502, Japan; s3igimi@nodai.ac.jp

**Keywords:** Human Papillomavirus (HPV), therapeutic vaccine, mucosal immunity, Cervical Intraepithelial Neoplasia 2 (CIN2), cervical cancer, lactobacillus-based vaccine

## Abstract

Cervical intraepithelial neoplasia (CIN), a precursor lesion to cervical cancer, is caused by high-risk human papillomavirus (HPV); high-grade CIN lesions (CIN2-3) are precancerous and require treatment. No globally approved therapy is available for CIN2-3 treatment. This study is a placebo-controlled randomized clinical trial of GLBL101c treatment for CIN2 in 40 patients with HPV16-positive CIN2 who were 1:1 randomized to receive GLBL101c (1 g/daily) or placebo for 5 days at 1, 2, 4, and 8 weeks. No differences were noted between the GLBL101c and placebo groups for patient background and adverse events. Moreover, no statistically significant difference was noted between the two groups at the primary endpoint, pathological regression after 16 weeks of the first oral dose; however, only in the GLBL101c group, two patients had complete regression (CR; regression to normal within 16 weeks). IFNγ production was significantly correlated with the number of spots identified by the interferon gamma enzyme-linked immunospot (IFNγ-ELISPOT) assay using cervical lymphocytes (CxLs) or peripheral blood mononuclear cells. In the two cases of CR, E7-specific Th1 immune responses were observed at week 16. Therefore, we concluded as a novel *Lactobacillus*-based vaccine with stronger immunogenicity than GLBL101c should be developed.

## 1. Introduction

Cervical cancer is the cause of more than 300,000 deaths worldwide each year [1]. Approximately 99% of cervical cancer cases are associated with genital infection with high-risk human papillomaviruses (HR-HPVs). Cervical cancer is most commonly associated with HPV type 16 (HPV16), followed by HPV types 18, 31, 33, 35, 39, 45, 52, 56, 58, and 68 [2,3]. Although it is expected that HPV vaccines will be able to eradicate cervical cancer in the future, the HPV vaccination rate is currently limited in many countries and regions due to cost issues; therefore, it will take a long time to eliminate cervical cancer globally [4]. Therefore, the development of therapeutics to treat precursor lesions (such as cervical intraepithelial neoplasia (CIN)) is essential even though HPV vaccination programs have been implemented worldwide. Surgical resection is the only treatment for early cervical cancer and precancerous cervical lesions (CIN2-3), which peak in women in their 20s and 30s. Currently, no pharmacological treatments are available. Hysterectomy leads to infertility, and conization of the cervix worsens obstetrical outcomes in subsequent pregnancies. Therefore, conization is considered as a standard surgical treatment of CIN2-3 especially for patients who is willing to preserve their fertility; however, the risk of preterm birth and the rate of cesarean delivery and low birth weight are approximately three times higher after conization [5,6,7]. Because the age at which CIN2-3 develops coincides with the age at which a woman becomes pregnant and gives birth, the worsening of obstetrical outcomes because of cervical incompetence after partial resection of the cervix is the major issue for reproductive health of young women. Therefore, the development of a therapeutic agent for CIN as a non-surgical treatment for CIN2-3 is an unmet medical requirement.

HPV E6 and E7 oncoproteins are essential for the maintenance of the neoplastic phenotype because E6 and E7 induce dysfunction of tumor suppressor genes such as *TP53* and *RB1*, respectively [2]. The ubiquitous and overexpression of HR-HPV oncoproteins E6 and E7 in cervical epithelial cells results in progression of CIN2-3 to cervical carcinoma. E6 and E7 are essential viral proteins for the progression of CIN to cervical cancer and maintenance of HPV-associated cancer [2]. E7 is known to be highly immunogenic in humans, whereas E6 is less likely to induce immune responses in humans. Therefore, HR-HPV E7 is not only a viral protein, but also a tumor antigen of HPV-associated cancer, suggesting that E7 is the most definitive tumor antigen and the target molecule of immunotherapy in the case of HPV-associated cancers such as cervical cancer.

Previous prospective cohort studies on the natural history of CIN have demonstrated that CIN regresses spontaneously by host immune responses. In one study, spontaneous regression of CIN was observed in approximately 70% of patients with CIN1 and 50–60% of patients with CIN2 during 2 years of follow-up [8]. Another study reported that approximately 20% of CIN3 regressed within 2 years of follow-up with no intervention [9]. Recognition of HPV E7 by host immune cells results in Th1 immune responses followed by immunological clearance. This process of spontaneous regression may be used to develop a novel noninvasive therapeutic strategy for CIN treatment, referred to as cancer immunotherapy or HPV therapeutic vaccine. Several clinical trials (phase I–III trials) of various HPV therapeutic vaccines have been conducted for the treatment of CIN2-3 since the 1990s, the majority of which have used HPV E7 as the target molecule [10]. This indicates immunotherapy targeting HPV E7 is a promising therapy to treat CIN based on its natural history. In earlier trials, vaccine antigens were administered either intramuscularly or subcutaneously to induce E7-specific cell-mediated immunity (E7-CMI) in immunized patients. However, their immune responses have not always correlated with clinical efficacy and none have been applied clinically till date. Recently, Trimble et al. reported on the effect of intramuscular administration of plasmid DNA vaccine, VGX-3100, to 167 patients with HPV16-positive CIN2-3 in a randomized placebo-controlled trial [11]. Regression to normal was observed in 48% of the VGX-3100 group patients and 30% of the placebo group patients; thus, a significantly higher regression rate was observed in the VGX-3100 group. However, in this trial, 30% of the patients with CIN2 were enrolled in the VGX-3100 group and 26% in the placebo group. Because compared with CIN3, CIN2 is likely to regress spontaneously, the difference in patients’ background might influence the result. Although a significant difference in clinical efficacy was noted, adverse events at the inoculation site occurred in 98% of the patients due to intramuscular injection. Moreover, VGX-3100 is currently in a phase III trial in the US; however, nothing is currently in clinical usage.

CIN lesions develop in the cervical mucosa; therefore, local mucosal lymphocytes possessing E7-CMI in the cervix are likely to play a direct role in immunological clearance of CIN lesions. We hypothesized that mucosal immunity to HPV E7 should be induced to immunologically eliminate the mucosal lesion. In the mucosal immune system, the Peyer’s patches (gut-associated lymphoid tissues (GALT)) or mesenteric lymph nodes are known to be the inductive sites for the genital mucosa (including the cervical mucosa). Gut-derived mucosal lymphocytes were recruited and activated at GALT and mesenteric lymph nodes, which are home to the genital mucosa, through peripheral blood. The mucosal lymphocytes have a unique surface antigen called integrin β7, which binds to its natural ligands (MadCAM) expressed on the endothelial cells of the mucosal vessels and infiltrates the mucosa. In addition, the integrin β7 binds to E-cadherin expressed on the cervical epithelium, and mucosal lymphocytes may accumulate on the epithelium. We previously demonstrated that approximately 20–40% of CD3+ T cells in the cervical epithelium of patients with CIN were gut-derived T cells expressing integrin β7+; furthermore, we found that CIN was more likely to regress when the number of T cells expressing integrin β7+ was high [12]. Thus, we considered that memory helper and killer T cells educated in the gut mucosa can infiltrate into CIN2-3 lesions, and Th1 cells activated by HPV E7 antigen will recognize CIN2-3 cells and induce Th1 immune responses to eliminate the lesion. We have generated an oral therapeutic vaccine ‘‘GLBL101c”, an attenuated HPV16 E7-expressing *Lactobacillus casei* [13,14,15].

After these pre-clinical studies, we conducted an exploratory phase I/IIa clinical trial. Patients with CIN3 lesions positive for HPV16 were administered GLBL101c once daily for 5 days at weeks 1, 2, 4, and 8. None of the 17 patients had grade ≥ 2 adverse events. Moreover, in all 17 patients, none of the grade 1 adverse events were causally related to GLBL101c. The regression rate of CIN3 to CIN2 at 9 weeks of treatment initiation was 80%, and the regression rate of CIN3 to CIN1/normal in the first 12 months of treatment was 38.4%, which was clearly higher than the rate of spontaneous regression (approximately 10% per year). In addition, compared with the group with no regression of CIN lesion, the group with regression of CIN lesion to CIN2 or less clearly had a higher induction of E7-specific mucosal IFNγ-producing cells into cervical intraepithelial lymphocytes [15].

In this study, we aimed to explore the efficacy and adverse event of GLBL101c in patients with CIN2 in a randomized controlled setting. CIN2 is likely to spontaneously regress [8], and thus the placebo-setting was required to verify the clinical efficacy of GLBL101c. Patients with CIN2 were mainly enrolled in the placebo group to reduce the risk of progression to invasive cancer.

## 2. Patients and Methods

### 2.1. Patients

We enrolled patients with histologically confirmed ectocervical CIN2 lesions and HPV16 infection only (i.e., without other HR-HPV type infection) as documented using the in-house PGMY-CHU HPV genotyping method, which can detect 34 HPV subtypes [15]. Other eligibility criteria were as follows: age: 20–45 years, colposcopic evidence of a persistent high-grade lesion after 4 weeks of biopsy, normal pretreatment laboratory blood values, and having signed the informed consent.

All the enrolled patients were required to provide written informed consent. Exclusion criteria were as follows: (1) any signs of invasive disease; (2) pregnancy or lactation; and (3) HIV positivity, presence of an immunosuppressive disease, or use of immunosuppressive medications.

### 2.2. Study Design

The spontaneous regression rate of CIN2 in the 2-year follow-up period was thought to be 50–60% [8]. Thus, this study was designed as a placebo-controlled randomized trial. Patients were randomized into two groups considering their age and follow-up duration for CIN. In our previous phase I/IIa dose-escalation clinical trial, we found that the optimal dose of GLBL101c was 4 capsules daily (250 mg/capsule) [15]. All patients received four rounds of oral vaccination at 1, 2, 4, and 8 weeks. One dose of oral GLBL101c was administered every morning after fasting for 5 days in each treatment week as in our previous phase I/IIa clinical trial [15]. Patients were treated only GLBL101c or placebo during this trial. We followed our subjects for 16 weeks prior to reassessment and possible treatment to ensure optimal patient safety.

The primary endpoints were to evaluate the safety and pathological efficacy of vaccination at week 16. Grading of CIN lesions was independently performed by several blinded experienced pathologists using strict criteria [16]. The criteria used for evaluation were (i) complete response (CR), no abnormal findings; (ii) partial response (PR), regression to CIN1; (iii) stable disease (SD), no change from CIN2; and (iv) progression of disease (PD), progression from CIN2 to CIN3 or carcinoma [17]. Histological and cytological specimens were collected under colposcopic guidance. Biopsies from the same site were obtained at baseline and week 16. If CIN3 had downgraded to CIN2 or less, further surgical intervention was averted. Otherwise, patients with CIN3 underwent cervical conization or laser ablation.

Secondary endpoints were evaluated at week 16 by assessing cytological efficacy, induction of E7-specific Th1 immune response in cervical lymphocytes (CxLs) and peripheral blood mononuclear cells (PBMCs), and adverse events. Cytological findings were evaluated as follows: (i) better, regression into negative for intraepithelial lesion or malignancy (NILM) or LSIL; (ii) stable, no change from high-grade squamous intraepithelial lesion (HSIL); and (iii) worse: progression to squamous cell carcinoma (SCC) [15].

All vaccinations were performed between February 2014 and March 2017. The study was sponsored by the Project Promoting Clinical Trials for Development of New Drugs from Japan, Agency for Medical Research and Development (AMED), Japan, and approved by the Medical Ethics Committee of the University of Tokyo, Faculty of Medicine. All patients provided written informed consent. All data underwent independent third-party management and analysis and were evaluated by a third-party committee for efficacy and safety.

### 2.3. Composition of the Vaccine

GLBL101c, provided by ANGES Inc. (Osaka, Japan), was generated from a heat-attenuated recombinant *L. casei* expressing mutated HPV16 E7 as described previously [13,14]. Briefly, the HPV16 E7 gene was modified by inserting point mutations in the Rb-binding site [13]. The attenuated *L. casei* was purified and dried into powder. The E7 protein content in each lot of GLBL101 powder was confirmed by Western blot analysis. A capsule designed to degrade in the bowel was filled with 250 mg of GLBL101c powder.

### 2.4. Collection and Processing of Cervical Specimens

PBMCs and CxLs were sampled from enrolled patients at pre-vaccination and weeks 9 and 16. CxLs were collected using a Digene cytobrush as described previously [10]. The cytobrush was placed into a 15 mL tube containing culture media. After incubating the sample with 5 mM DL-dithiothreitol, the floating cells were collected by centrifugation and resuspended in Percoll. PBMCs and CxL-derived mononuclear cells were purified by centrifugation with Percoll and washed with phosphate-buffered saline (PBS). Thereafter, all samples were frozen at −150 °C until use.

### 2.5. Safety and Tolerability

Clinical assessments, laboratory testing, and adverse event monitoring were conducted at weeks 9 and 16. Adverse events were graded using the Common Terminology Criteria for Adverse Events (CTCAE) version 4.0. CTCAE grades adverse events on a scale of 1 to 5, with higher grades indicating greater severity.

### 2.6. Immunological Responses to HPV16 E7

Frozen PBMCs and CxLs were used for modified interferon-gamma (IFN-γ) enzyme-linked immunospot (ELISPOT) assay as described previously [18]. Briefly, 1 × 10^5^ PBMCs or 1 × 10^4^ CxLs were added to each well and cultured with 10 µg/mL of 15-mer overlapping peptides (overlapping by 10 amino acids spanning HPV-E7). In the case of CxLs, 1 × 10^5^ PBMCs sampled in the pre-vaccination week were added to each well as antigen-presenting cells. After 18 h of incubation, the cells and media were harvested from the ELISPOT plate, transferred to a 96-well round-bottom plate, and cultured for more 30 h to measure IFN-γ concentration in the supernatant. The concentration of IFN-γ was quantified using the BDTM Cytometric Beads Array Human IFN-γ Enhanced Sensitivity Flex set (Becton Dickinson, Mountain View, CA, USA) according to the manufacturer’s instructions.

### 2.7. Statistical Analysis

Based on our explanatory study, we assumed that the regression rate of CIN3 to CIN2 was approximately 30% for the placebo group and that for the GLBL101c group was 70% [15]. A sample size of 15 patients in each group was required to achieve a detection power of 80% and a two-sided alpha level of 0.05. Therefore, we recruited a total of 40 participants.

For safety analysis, we calculated *p* values to compare the difference between the proportions of adverse events in each group using the Fisher’s exact test. We assessed efficacy based on clinical responses evaluated by histological examination at week 16 and compared the proportions of CR/PR/SD/PD and cytological findings between the two groups using the Mantel test.

## 3. Results

### 3.1. Study Population and Adverse Events

A total of 40 patients who were histologically diagnosed with CIN2 and were positive for HPV16 alone as determined by the PGMY-CHU HPV genotyping assay were randomized into two groups: GLBL101c and placebo considering patients’ age and CIN follow-up duration (Supplementary Figure S1). One of the 20 patients from each group withdrew from the study because of pregnancy. Thereafter, 19 patients from each group were analyzed for primary and secondary endpoints. Characteristics of participants are summarized in Table 1. No difference was noted between the two groups for patient characteristics such as patient age and follow-up duration (Table 1). Adverse events observed in each group are listed in Table 2. No patient experienced severe adverse events in each group. No significant difference was found between two groups for each event (Table 2). The adverse events were graded as grade 1 or 2 according to CTCAE ver.4. No patient withdrew from the study because of adverse events or progression of their disease.

### 3.2. Clinical Responses to Administration

In this study, the clinical responses to GLBL101c were evaluated by histological examination at week 16. Pathological findings of the GLBL101c and placebo groups are summarized in Table 3. In the placebo group, no CR (regression to normal) was observed in any patient, whereas PR (regression to CIN1) was observed in seven (37%) patients. In the GLBL101c group, CR was observed in two (11%) patients and PR was observed in two (11%) patients. The rate of CR or CR+PR did not significantly differ between the two groups. The primary endpoint was evaluated at week 16, which was only 2 months after the final dose of GLBL101c. Nevertheless, pathological CR was observed in two GLBL101c group patients but not in any placebo-group patients. A previous cohort study reported that HPV16/18-related CIN1-2 spontaneously regressed to normal at a rate of 66% in 2 years and the time to regression to normal was 7.7 to 22.7 months (median time: 16.3 months) [6]; therefore, the two patients administered with GLBL101c had a rapid regression, which had a different clinical course from spontaneous regression to CIN1 in the placebo group. Cytological evaluation revealed that there was no difference in the clinical response at week 16 between the GLBL101c and placebo groups (Table 3).

### 3.3. Correlation between E7-Specific IFNγ-Producing Cells and IFNγ Secretion

**Figure 1:**
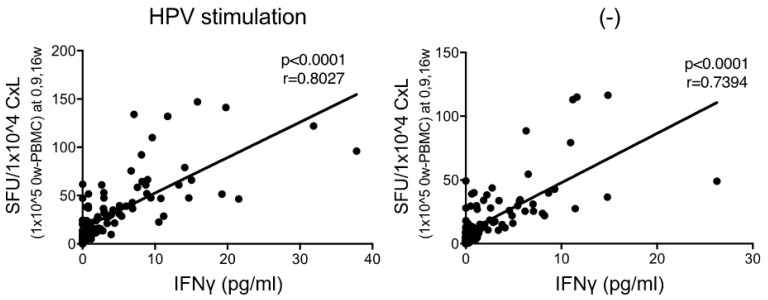
Correlation between the number of spots on ELISpot and IFNγ production for E7-specific Th1 response in CxL. To confirm the Th1 feature of the CxL, the number of spots on ELISpot was plotted on the Y-axis and the amount of IFNγ (pg/mL) on the X-axis, and the correlation was examined. Regardless of lymphocytes with or without E7 stimulation, a higher number of spots correlated with higher IFNγ production, indicating that ELISpot can be used to measure E7-specific Th1 immune responses in CxL.

In our previous clinical trial, we collected CxLs from patients with CIN and measured the number of IFNγ-producing cells using the ELISPOT assay to investigate the E7-specific Th1-type immune response in local cervical lesions [15]. GLBL101c exerts pharmacological effects through mucosal immunity, and therefore we considered that this evaluation system of mucosal immune responses (IFNγ-ELISPOT assay using CxLs) is the direct method. However, CxLs have a disadvantage in that the number of lymphocytes that can be collected varies due to individual differences in cervical mucus secretion and changes in the composition of immune cells depending on the time of collection (i.e., the time of the menstrual cycle) [12]. Therefore, an evaluation system using PBMCs, in which the number of lymphocytes does not vary individually, was implemented in this clinical trial. In addition to the number of IFNγ-producing cells in CxLs, we examined the amount of IFNγ secreted from CxL by E7-peptide stimulation. CxLs were collected from patients at week 9 or 16 regardless of whether in the GLBL101c or placebo group. Figure 1 demonstrates the correlation between the numbers of IFNγ-producing cells determined by the ELISPOT assay and the amount of IFNγ (protein) secreted from CxLs. This confirmed that the ELISPOT assay used in this trial reflected IFNγ production (Th1 immune response) and that IFNγ production was not detected only in PBMCs, although PBMCs were used as antigen-presenting cells in the ELISPOT assay.

### 3.4. E7-Specific Th1 Immune Responses to Administration

**Figure 2:**
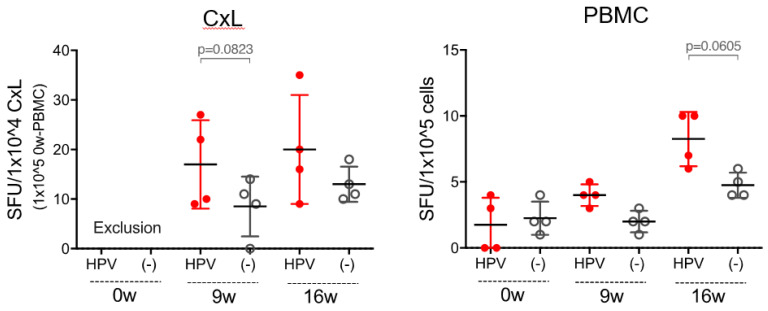
Time-dependent increases in the number of IFNγ-producing cells in the GLBL101c group. The number of IFNγ-producing cells at pre-immune (0 week), 9 week, and 16 week was compared for CxL and PBMC with (red dot) and without (circle dot) E7 stimulation. The number of spots with E7 stimulation (HPV+) tended to increase in a time-dependent manner, with three samples showing a similar increase.

We measured CxLs and PBMCs collected at baseline and at weeks 9 and 16 from patients who participated in the trial using the ELISPOT assay. Figure 2 depicts the ELISPOT assay data of a representative patient; in this patient, the number of E7-specific IFNγ-producing cells increased after GLBL101c administration. Oral administration of GLBL101c trended to elicit an increase in E7-specific IFNγ-producing cells in CxLs and PBMCs at weeks 9 and 16 in this patient, although the difference between E7 stimulation and no stimulation had marginal significance (Figure 2). Notably, this patient experienced a regression to normal 12 months after administration, although the outcome was SD at week 16.

In this study, positive E7-specific Th1 immunoreactivity was defined as a significant increase in the number of spots upon stimulation with E7-overlapping peptides compared with non-stimulation (Table 3). An increase in E7-specific Th1 immune response in either CxLs or PBMCs was found in 10 (52.6%) of 19 patients in the GLBL101c group and seven (36.8%) of 19 patients in the placebo group using the ELISPOT assay. Although no statistical difference was noted in the induction of E7-specific Th1 immune response between the GLBL101c and placebo groups, >50% of the patients administered with GLBL101c had a significant increase in immunological response to GLBL101c, which is consistent with the findings of our previous clinical study of CIN3 treatment [15].

### 3.5. Characteristics of Patients with Rapid Regression to Normal

**Figure 3:**
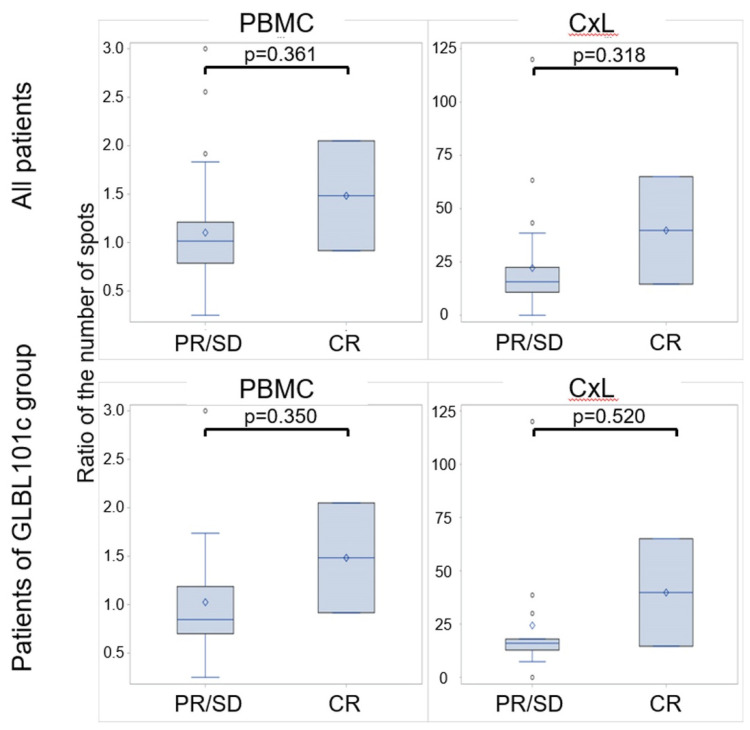
Ratio of the increase in the number of spots with to without E7 stimulation, comparing CR and PR/SD cases. The ratios for all patients (upper panels) or patients of GLBL101c group (lower panels) were plotted for PBMC (left panels) or CxL (right panels). Plot means each case. Box and bars mean 25–75% of all ratios and median and 1-SD, respectively. E7-specific Th1 immune response tended to be higher in CR patients in both PBMC and CxL when compared with PR/SD patients.

Thereafter, we focused on the correlation of E7-specific Th1 immunity and clinical rapid regression to normal (CR) within 4 months. In comparison between patients with CR versus patients with PR or SD, E7-specific Th1 immune response in patients with CR trended to be higher than that in patients with PR or SD. The ratio of increase in the number of E7-specific IFNγ-producing cells with E7 stimulation versus without stimulation is presented in Figure 3. E7-specific Th1 immune response in both PBMCs and CxLs tended to be higher in patients with CR, regardless of GLBL101c or placebo administration (Figure 3, upper panel). Moreover, this trend was observed in patients administered GLBL101c, suggesting that patients with strongly induced E7-specific Th1 immune responses were likely to have a rapid regression (Figure 3, lower panel).

**Figure 4:**
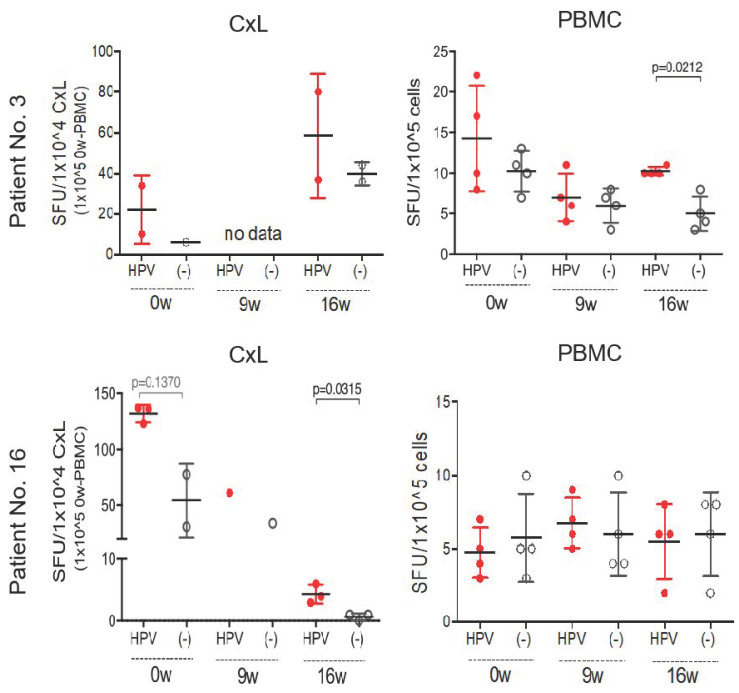
Increase in the number of IFNγ-producing cells in the patients with CR. The number of spots of CxL and PBMC at pre-immune (0week), 9 week, and 16 week was shown for patients 3 and 16 of CR cases that regressed to normal within only 4 months. In both cases, at week 16, E7 stimulation (HPV+; red dot) was significantly elevated compared to no E7 stimulation although the number of spots without stimulation (black dot) varied from specimen to specimen.

The ELISPOT data for all cases with clinical CR are presented in Figure 4. In patient number 3 (upper panels), the number of CxLs was not sufficient to provide accurate data; however, E7-specific IFNγ-producing cells in PBMCs were significantly elevated at 16 weeks (*p* = 0.0212). In patient number 16, a sufficient number of CxLs was obtained and E7-specific IFNγ-producing cells in CxLs were significantly elevated at 16 weeks (*p* = 0.0315), whereas no elevation was observed in PBMCs. The number of spots with E7 stimulation had increased at 0 week (pre-immune) in PBMCs of patient number 3 and in CxLs of patient number 16; however, this increase was not significant considering the high background (without stimulation) of ELISPOT (Figure 4). Interestingly, both patients belonged to the grouped treated by GLBL101c. In patients with rapid regression, E7-specific Th1 immune response was found to be induced clearly after oral administration with GLBL101.

## 4. Discussion

In this placebo-controlled randomized clinical trial, we investigated the efficacy of GLBL101c on CIN2. The clinical efficacy of GLBL101c on CIN2 was found to be limited. However, all patients with rapid regression (CR) at week 16 were administered GLBL101c, and E7-specific Th1 immune responses were elicited in these patients, suggesting that immune responses to GLBL101c can result in clinical remission of CIN2.

Our statistical analysis revealed that the clinical and immunological responses did not significantly differ between the GLBL101c and placebo groups. Three major reasons for this finding were the small sample size, low expression levels of E7 in CIN2 lesion, and limited antigen-presenting by GLBL101c.

The rate of CR (regression to normal) in the GLBL101c group versus the placebo group was 11% (2/19 patients) versus 0% (0/19 patients), and the CR+PR rate, meaning the response rate in the GLBL101c group, was 22%. Based on the findings of our previous clinical trial, the expected response rate for GLBL101c was set at 70% to calculate the number of patients to be enrolled [14]. In the clinical trial for CIN3, clinical response (calculated as regression from CIN3 to CIN2 or less) was reported to be 70% (seven of 10 patients). The target diseases and endpoints were different in the previous and current trials; therefore, it may not be appropriate to determine the expected response rate for the current study by referring to the response rate of the previous study. We consider that due to the underestimation of the number of patients enrolled, a significant difference was not observed between the two groups for the CR rate.

It has been demonstrated that the positive rate of E7 expression in CIN increases with grade, both at the immunochemical [19,20] and mRNA levels [21,22]. The low positive rate of E7 expression in CIN2 compared with that in CIN3 suggests that E7-specific mucosal Th1 immune responses are less likely to occur in patients with CIN2. Therefore, the response rate to GLBL101c in this clinical trial was lower than expected. In this study, we did not measure the E7 expression level in the cervical lesion of each patient. Therefore, we could not examine the correlation between the E7 expression and the ability of GLBL to induce E7-specific immunity or clinical efficacy. Furthermore, Wang-Johanning et al. revealed that E7 expression level (i.e., copy number of E6/E7 mRNA) increases with CIN grade [22]. This suggests that the amount of E7 protein, the target antigen, is lower in CIN2 than in CIN3, and that local E7-specific immune responses at the cervix are less likely to be elicited. Overall, GLBL101c was able to provide a clinical effect against CIN3 through an E7-specific Th1 immune response [15]; however, it was difficult to induce an immune response sufficient to elicit a therapeutic effect against CIN2, which has low E7 expression. To elicit a therapeutic effect against CIN2, a therapeutic vaccine that can induce a stronger E7-specific Th1 immune response than GLBL101c through mucosal immunity is required.

It is well known that immunotherapy provides clear distinction between effective and ineffective cases. The pharmacological mechanism of GLBL101c is induction of mucosal immunity-mediated E7-specific Th1 immune response; therefore, the efficacy of GLBL may depend on individual differences in the ability to induce mucosal immunity to E7. Whether an E7-specific immune response occurs, and when it occurs, varies from patient to patient. A cohort study has demonstrated that the time to regression from CIN2 to normal in spontaneous regression was 7.7–22.7 months (median time: 16.3 months) [8]. In this study, we set week 16 (4 months after the first administration) as the endpoint. In our study, two patients had CR (regression from CIN2 to normal within 4 months), which strongly suggested that the regression was due to E7-specific immune response elicited by GLBL101c. Indeed, our immunological assay showed that these two cases had significant increase of E7-specific Th1 immune response.

In this study, we used CxLs for local immune response, PBMC for systemic immune response, and expanded PBMCs by amplifying small amount of E7-specific immune cells. In some cases, the number of CxLs was extremely small for analysis. In Figure 3, we calculated the ratio of spots with and without E7 peptide stimulation and illustrated the ratio on the graph. By contrast, the absolute number of spots in the ELISPOT assay using CxLs and PBMCs with and without E7 stimulation are compared in Figure 2 and Figure 4. The basal values without E7 stimulation varied between high and low weeks. Moreover, a significant difference in the absolute number of spots indicated that the patient had a significantly elevated number of E7-specific Th1 immune cells, which was observed for two patients with CR at week 16 (Figure 4).

As is shown, we described our result showing no significant clinical response by GBLB101c. As cases achieve CR showed significant immune response, we concluded the necessity of the study protocol modification to develop a new *Lactobacillus*-based vaccine that can induce a stronger Th1 response to E7, increase the number of patients, and lengthen the follow-up period in the next randomized controlled trial. We have already succeeded in developing a new type of an HPV16 E7-expressing *Lactobacillus*-based vaccine, IGMKK16E7, which can elicit a 4-fold greater E7-specific mucosal immunity than GLBL101c [23]. Furthermore, we have started a phase I/II placebo-controlled randomized controlled trial in 164 patients with HPV16-positive CIN2-3 (clinical trial registration number: UMIN000034253, jRCT2031190034) [24]. The design of the ongoing clinical trial has been modified based on the findings of this study, and the final analysis is planned in 2022. As mentioned, current standard therapeutics for CIN2-3 is conization. This series of clinical studies will bring us one step closer to commercializing a therapeutic agent for CIN2-3 treatment, an unmet need, and will greatly benefit patients with CIN as a treatment option.

## 5. Conclusions

In this clinical trial, the clinical and immunological responses did not significantly differ between the GLBL101c and placebo groups. We concluded as a novel *Lactobacillus*-based vaccine with stronger immunogenicity than GLBL101c should be developed

## Figures and Tables

**Table 1:** Characteristics of participants in this study.

Characteristics	GLBL-101c	Placebo
Number of patients	20	20
Age		
<30	5	3
≥30	15	17
Height	158.6	161.2
Weight (Median)	50.2	51.6
Initial blood pressure (Median)		
Max	115.4	112.4
Min	72.4	72.6
History of chlamydia infection	3	5
Current chlamydia infection	0	0
HIV antibody positive	0	0
Cytological finding		
NILM	1	4
LSIL	2	1
HSIL	11	13
N/A	6	2
* History of previous treatment		
YES	2	5
NO	17	15
N/A	1	0
Follow up duration		
<3years	14	15
≥3years	6	5

* Treatment for CIN lesion was laser vaporization in all patients.

**Table 2:** Adverse events.

Events	GLBL101c (*N* = 20)			Placebo (*N* = 20)			
	No. of Patients	(%)	No. of Events	No. of Patients	(%)	No. of Events	* *p*-Value
Constipation	0	0	0	0	0	0	-
Abdominal pain	2	10	2	3	15	3	1.000
Nausea	1	5	1	0	0	0	1.000
Palpitation	0	0	0	0	0	0	-
Headache	4	20	6	1	5	1	0.342
Peripheral neuropathy	0	0	0	1	5	1	1.000
Itch	1	5	1	0	0	0	1.000
Skin rash	2	10	2	0	0	0	0.487
The others	7	35	13	6	30	12	1.000
Total	17	85	25	11	55	17	0.082

* Fisher’s exact test.

**Table 3:** Clinical and immune responses.

Characteristics	Outcome (No. of Patients)				Total	*p*-Value
Histology at Week 16	CR	PR	SD	PD		
GLBL101c	2	2	12	3	19	0.344 *
Placebo	0	7	12	0	19	
Cytology at week 16	NILM	LSIL	HSIL	SCC		
GLBL101c	3	2	12	0	17	0.236 *
Placebo	7	1	10	0	18	
E7-specific immune response	Positive (9 weeks, 16 weeks)		Negative			
GLBL101c	10 (4, 6)		9		19	0.514 **
Placebo	7 (3, 4)		12		19	

*: Mantel test; **: Fisher’s exact test.

## Data Availability

No new data were created or analyzed in this study.

## Supplementary Materials

The following are available online at https://www.mdpi.com/article/10.3390/vaccines9040329/s1, Figure S1: Trial profile. Forty patients with CIN2 positive for HPV16 alone were enrolled and randomized; one patient each in the GLBL101c and placebo groups with a known pregnancy during protocol period was excluded from the analysis. The remaining 19 patients who completed the protocol were included in the analysis.

## Clinical Trial Registration

This study is registered with University Hospital Medical Information Network Clinical Trials Registry (UMIN-CTR; registration ID: UMIN000001686 [6 May 2009]), which is approved by the International Committee of Medical Journal Editors (ICMJE).
