# Supplementary material for: A Placebo-Controlled, Double-Blind Randomized (Phase IIB) Trial of Oral Administration with HPV16 E7-Expressing Lactobacillus, GLBL101c, for the Treatment of Cervical Intraepithelial Neoplasia Grade 2 (CIN2)
Source: Vaccines (Basel). 2021 Apr 1;9(4):329. doi: 10.3390/vaccines9040329 (PMC8066592; doi:10.3390/vaccines9040329)
Supplement: Supplementary file 1 [file vaccines-09-00329-s001.pdf]

vaccines

Supplementary Material

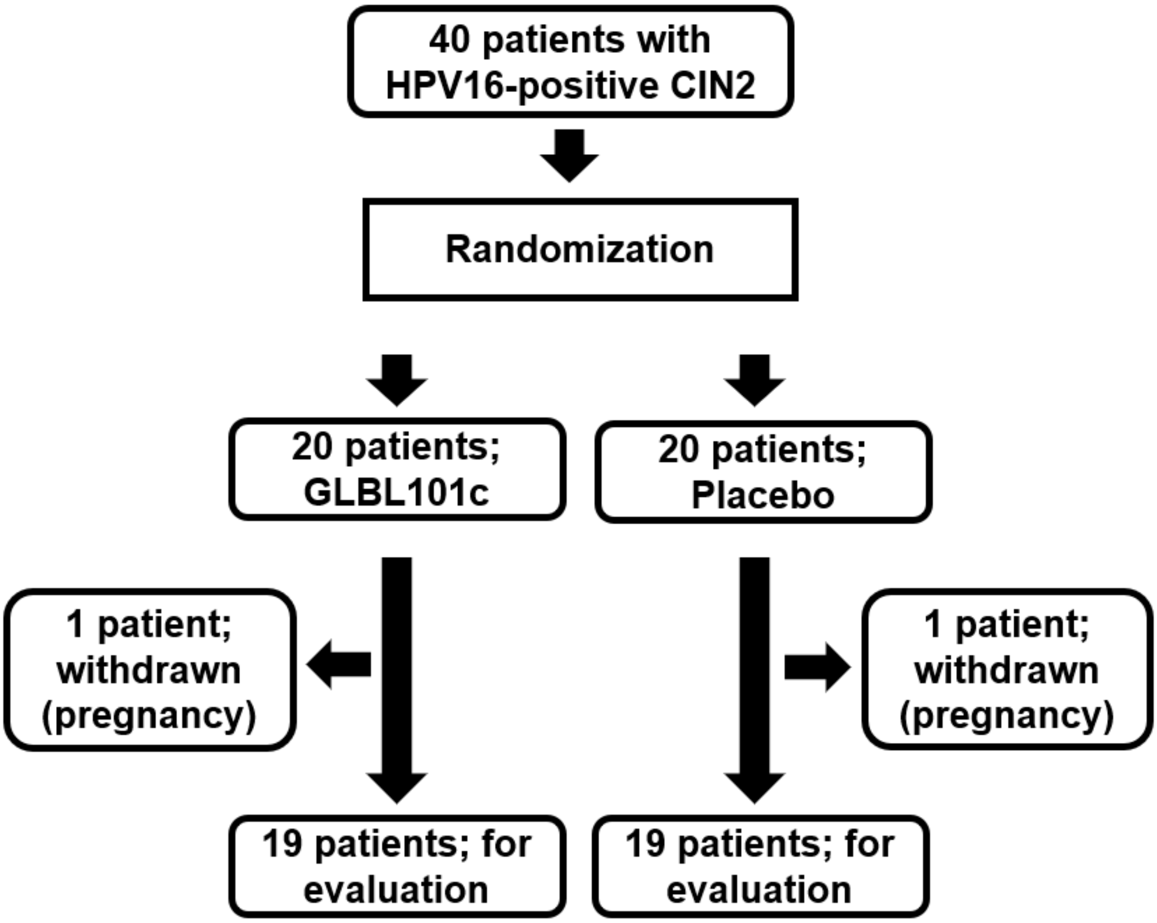

Figure 1. Trial profile. Forty patients with CIN2 positive for HPV16 alone were enrolled and randomized; one patient each in the GLBL101c and placebo groups with a known pregnancy during protocol period was excluded from the analysis. The remaining 19 patients who completed the protocol were included in the analysis.
